# New Fast Arctangent Approximation Algorithm for Generic Real-Time Embedded Applications

**DOI:** 10.3390/s19235148

**Published:** 2019-11-25

**Authors:** Mohieddine Benammar, Abdulrahman Alassi, Adel Gastli, Lazhar Ben-Brahim, Farid Touati

**Affiliations:** 1 Department of Electrical Engineering, Qatar University, Doha 2713, Qatar; adel.gastli@qu.edu.qa (A.G.); brahim@qu.edu.qa (L.B.-B.); touatif@qu.edu.qa (F.T.); 2 R&D Laboratory, Iberdrola Innovation Middle East, Doha 210177, Qatar

**Keywords:** arctangent approximation, position sensors, signals processing, CORDIC, look-up-tables, minimax optimization, novel algorithm, rational approximations

## Abstract

Fast and accurate arctangent approximations are used in several contemporary applications, including embedded systems, signal processing, radar, and power systems. Three main approximation techniques are well-established in the literature, varying in their accuracy and resource utilization levels. Those are the iterative coordinate rotational digital computer (CORDIC), the lookup tables (LUTs)-based, and the rational formulae techniques. This paper presents a novel technique that combines the advantages of both rational formulae and LUT approximation methods. The new algorithm exploits the pseudo-linear region around the tangent function zero point to estimate a reduced input arctangent through a modified rational approximation before referring this estimate to its original value using miniature LUTs. A new 2nd order rational approximation formula is introduced for the first time in this work and benchmarked against existing alternatives as it improves the new algorithm performance. The eZDSP-F28335 platform has been used for practical implementation and results validation of the proposed technique. The contributions of this work are summarized as follows: (1) introducing a new approximation algorithm with high precision and application-based flexibility; (2) introducing a new rational approximation formula that outperforms literature alternatives with the algorithm at higher accuracy requirement; and (3) presenting a practical evaluation index for rational approximations in the literature.

## 1. Introduction

Efficient and fast arctangent approximation is utilized in various applications ranging from signal processing, sensors, and measurements to large-scale power systems [1,2,3,4]. For instance, some relay models in power systems continuously utilize fast arctangent approximations for monitoring phase angles and making switching decisions based on the measured phase shifts [2]. On the other hand, sinusoidal encoders (position sensors) provide electrical signals related to the sine and cosine values of their mechanical shaft angle *θ*; and conventional ratiometric converters associated with these devices employ the tangent/cotangent method to decode the signal and obtain the position angle through arctangent approximation [3,5]. More recently, arctangent demodulators have been used as system components in healthcare monitoring, or remote vital sign detection through Doppler radar systems [6,7]. Consequently, the aim of this paper is to present and validate a new real-time arctangent approximation algorithm with enhanced performance and capabilities, facilitated by rapid advancements in micro-embedded chip technologies.

The arctangent function atan(u) is defined for all real numbers *u*. Direct, single input, atan(u) implementation is used to estimate unknown input angles in the 1st and 4th quadrants, whereas most embedded applications aim to estimate full-range (360°) angles using the atan2(u) function with inputs of complex forms z=I+iQ, where the pair (I, Q) represents the in-phase *I =* cos(θ) and quadrature *Q =* sin(θ) components [8,9,10].

**Figure 1:**
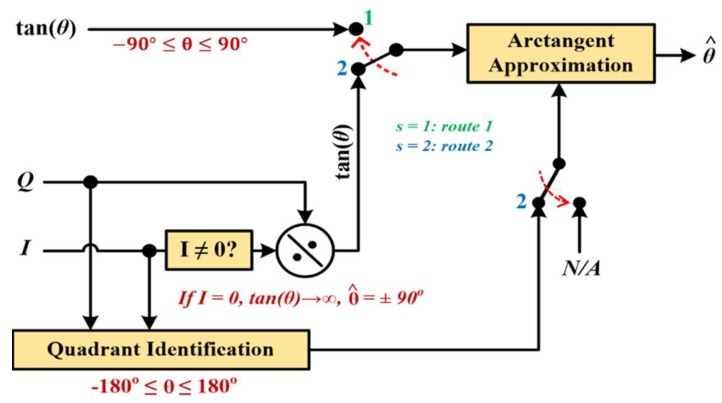
Generic arctangent approximation block diagram.

A generic block diagram for arctangent approximation is shown in Figure 1 to classify the different possible estimations based on their available set of inputs, where the estimated angle $\hat{\theta }$ is calculated within the range (−90° to 90°) for a single input signal (rarely used), and (−180° to 180°) when (*I, Q*) inputs are accessible. In this work, the true arctangent is referred to as θ while the estimated arctangent is denoted $\hat{\theta }$.

Taylor series infinite expansion of atan(u) has been conventionally used for angle estimation [11]. However, the large number of required terms to achieve high accuracy is deemed impractical for many real-time applications due to the high required execution time and variable hardware resource requirements [1]. Thus, different algorithms have been presented in the literature for this purpose. These are classified to three main categories: (1) iterative methods, which are mainly based on the coordinate rotational digital computer (CORDIC); (2) look-up-tables (LUTs)-based; and (3) rational formulae-based techniques [12].

The new arctangent approximation algorithm presented in this paper for the first time, combines the advantages of both LUT and rational techniques, while covering the full 360° implementation range and guaranteeing a very small estimation error. The contribution of this paper is summarized as: (1) introducing a new arctangent approximation algorithm with flexible, user-defined accuracy levels; (2) introducing a new rational approximation formula that outperforms published alternatives with the algorithm at higher accuracies; (3) presenting a practical evaluation index for the existing arctangent rational approximation formulae that can be used as a benchmark for their performance.

This manuscript is structured as follows: Section 2 presents a short review of the existing arctangent approximation techniques. Section 3 introduces a new second order rational arctangent approximation and compares it to state-of-the-art approximation expressions from literature. Section 4 describes in details the proposed approximation algorithm that incorporates the new approximation expression. Section 5 is dedicated to the assessment and experimental verification of the proposed algorithm performance. The paper is then summarized by concluding remarks.

## 2. Review of Arctangent Approximation Techniques

### 2.1. Iterative CORDIC

The iterative CORDIC algorithm uses a series of addition and bit-shift operations for two-input (I, Q) tangent arguments. However, its convergence time is highly dependent on the required accuracy since its error $\left(\varepsilon_{COR}=\;\hat{\theta }-\theta \right)$ relies on the number of iterations *N* and is given by Equation (1) in radians.

(1) $$\varepsilon_{COR}\leq \;atan(2^{-N+1})$$

CORDIC algorithm can theoretically be used to achieve very high accuracy levels based on the number of iterations [9]. It also requires minimal memory resources for its implementation, but requires long computational time for high-accuracy estimates due to its iterative convergence nature [12], which is a critical drawback in some real-time implementations. Therefore, an efficient implementation in terms of computational time and memory still attracts new research to achieve further improvements and optimizations to CORDIC performance [10,12,13].

### 2.2. Lookup Tables (LUT) Techniques

LUTs-based algorithms are faster when compared to conventional CORDIC [4,12], although they require significantly higher memory resources to accommodate the different possible output combinations for a given input range [12]. Interpolation techniques are typically employed to improve the accuracy for outputs falling between LUT entries [2]. The algorithm locates pre-stored boundaries $tan\left(\theta_{1}\right)$ and $tan\left(\theta_{2}\right)$ of the interval in which the input tan(θ) is located, as well as the corresponding boundary angles $\theta_{1}$ and $\theta_{2}$.

These are then used to estimate the arctangent using interpolation. However, the non-linearity of the arctangent function requires large LUT in order to reduce the interpolation error of estimating the arctangent. Variations of conventional LUT algorithms are also presented in [12] by means of initially estimating the arctangent through coarse approximations and then utilizing reduced-size LUT for error corrections, where an Field Programmable Gate Array (FPGA)-based implementation architecture is also introduced.

Recently, a new LUT technique variation was presented in [11], introducing a reduced-size LUT that is addressed by a transformed single variable for full-range arctangent approximation, as opposed to conventional two-variable indices. The new method is shown to perform well when compared to other LUTs-based competitors, especially when large LUT storage size is used.

### 2.3. Rational Approximations

Arctangent approximation through rational expressions is dependent on optimized rational formulae, whose error behavior is determined by a set of optimized constants. Through adequate optimization, these expressions can be made to closely match the highly non-linear arctangent function. Equation (2) depicts a generalized rational expression (ratio of two polynomials) for arctangent approximations. Typically, these formulas in their basic form have a radian output.

(2) $$\mathrm{atan}\left(u\right)\approx \phi_{n,d}\;\left(u\right)=\frac{P_{n}\left(u\right)}{D_{d}\left(u\right)}=\frac{\sum_{i=0}^{n}a_{i}u^{i}}{\sum_{j=0}^{d}b_{j}u^{j}}$$

where u is the input angle tangent (u=tan(θ) or u=Q/I) and may be replaced by its absolute value for some odd terms in $P_{n}\left(u\right)$ or $D_{d}\left(u\right)\;$[1,14], with *n* and *d* being the numerator and denominator degrees, respectively, and (*a_i_* and *b_j_*) are the polynomials coefficients that need to be optimized to match the rational expression to atan(u). Polynomial approximations are a special case of Equation (2) when the denominator degree is equal to zero. 

Most existing rational expressions in literature are constrained to the −1≤u≤1 input range to limit their errors (i.e., the –45°≤θ≤45° quadrant), since the tangent function non-linearity becomes accentuated beyond these boundaries. Extended angle ranges are easily obtained through tangent and cotangent properties. The objective of the coefficients optimization (*a_i_* and *b_i_*) is to minimize the angle estimation error function $\varepsilon_{R}$(*u*).

(3) $$\varepsilon_{R}\left(u\right)=\phi_{n,d}\;\left(u\right)-\mathrm{atan}\left(u\right)$$

Lagrange interpolation or minimax optimization algorithms are mostly employed to obtain *ϕ_n,d_*(*u*) constants [8]. The latter technique is adopted in this paper, where the aim, as the technique name suggests, is to minimize the maximum error in the considered range of input according to Equation (4).

(4) $$L=\underset{c}{\min}\left\{\underset{u_{i}<u<u_{f}}{\max}\left\{\varepsilon_{R}\left(u\right)\right\}\right\}$$

Here, *c* represents the constants to be optimized and the output *L* is a vector of optimized coefficients (*a_i_* and *b_j_*), where *u_i_ < u < u_f_* defines the optimization range (e.g., 0<u<1 for 0<θ<45° range). Computer-based toolboxes and extensive search routines are used to execute this optimization.

### 2.4. Approximation Techniques Qualitative Comparison

The aforementioned arctangent approximation methods vary in their level of accuracy and computational resource optimization. Appropriate technique selection is typically based on application constraints. Usually, the metrics used to evaluate approximations include (i) the input range; (ii) the target maximum approximation error; (iii) the execution time; and (iv) available memory and hardware resource limitations.

Some techniques provide more implementation flexibility in terms of adjustable accuracy versus execution time (e.g., CORDIC), whereas rational approximations provide constant maximum errors depending on the optimized formula. LUT methods, on the other hand, are advantageous in terms of their fast execution versus increased memory requirements. Table 1 summarizes qualitatively the main advantages and limitations of the three techniques.

## 3. Rational Formulae Comparison

Before introducing the new arctangent approximation algorithm in Section 4, it is important to address and classify rational approximation formulae, since they are essential to the algorithm implementation. Thus, existing rational approximations from the literature are presented and discussed here, in addition to introducing a new 2nd order arctangent approximation formula and comparing its performance to existing options.

The outcomes of this section can also be considered separately from the new presented algorithm, in terms of providing a practical index that systematically compares the performance of each surveyed approximation formula from the literature for the first time using a common implementation Digital Signal Processor (DSP) platform to compare their standalone execution time. The execution time is used as a metric in this context rather than the number of mathematical operations to simplify the comparison, because mathematical operations vary in their complexity (e.g., division is the most expensive in terms of the required hardware resources).

### 3.1. New 2nd Order Rational Approximation Formula

Many rational expressions, with d>0 (Equation 2), are derived based on an intuitive manipulation of the arctangent function’s Taylor series expansion by writing *u*/*D_d_* (*u*) ≈ atan (*u*) or *u* ≈ *D_d_* (*u*) × atan (*u*) where the Taylor series of the arctangent is used. A selection of suitable polynomial *D_d_* (*u*) with appropriate terms and coefficients can lead to acceptable approximations of the arctangent.

The approximation in [10] was based on this idea; however, the intuitive choice of the even power in the denominator led to a large residual error in the approximation. The alternative approximation in [15] makes use of odd and even powers but reverts to modifying the numerator as well; this does not lead to a noticeable improvement to the residual error and results in a slight increase in the computational time compared to that of [10].

On the other hand, the new second order rational expression that is introduced for the first time in this work aims to balance these factors. The new approximation in Equation (5) maintains odd and even powers in the denominator, while limiting the numerator to *u* only with significant reduction in maximum error compared to the approximations in [10] and [15]. This new expression of the arctangent approximation (in rad) is of the form

(5) $$\mathrm{atan}\left(u\right)\approx \frac{u}{1+b_{1}\left|u\right|+b_{2}u^{2}}.$$

The optimal coefficients *b*_1_ and *b*_2_ are obtained for the input range −1≤u≤1 using minimax optimization and are found to be $b_{1}=0.0443$ and $b_{2}=0.2310$, resulting into a maximum error of 0.0777°. The optimization is carried out for this interval here in order to have a common comparison ground to the other reported expressions in literature. 

### 3.2. Rational Formulae Classification

The maximum errors associated to the polynomial/rational approximations reported in the literature have been re-generated through MATLAB scripts over the same range −1≤u≤1 for verification, except for the approximation presented in [8] as it is derived for 0≤u<∞. Note that the work in [1] includes three different approximations. Thus, these are referred to hereafter as [1]-a, [1]-b, and [1]-c, respectively.

The execution time of each approximation has been calculated using a dedicated DSP platform. The used eZDSP-F28335 board runs at a speed of 150 MHz, utilizing a 32-bit floating-point unit. The board is also compatible with MATLAB/Simulink, enabling direct implementation of the models, which are developed using MATLAB/Simulink programming environment. The execution time of a given algorithm is measured by using one of the digital input/output pins of the eZDSP-F28335 platform to output a pulse whose high state begins once the tangent input is read by the algorithm and ends once the estimated arctangent is calculated/displayed.

**Figure 2:**
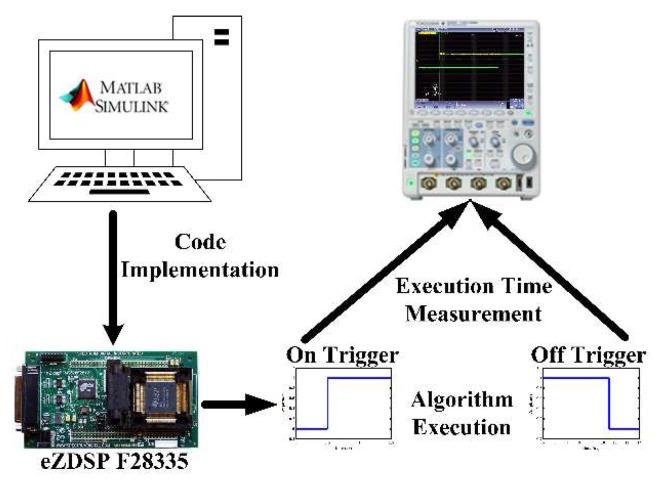
Practical verification setup block diagram using the eZDSP-F28335 platform.

The resulting execution times using this methodology are relatively high (few μs against a DSP clock speed of 150 MHz) because of the Simulink-based implementation. Having said that, the same platform is used throughout this work under the same conditions explained in Figure 2 in order to compare the results on a common-ground. It should also be noted that the consistency of the reported execution times in this paper was verified (i.e., the timing result of executing the same command multiple times is consistent).

The performance comparison of the various approximations over the same input range is summarized in Table 2, where the entries are ordered by their minimized maximum errors. The rational 4th order approximation presented in [14] provides the best error performance with a maximum error of 0.003°, while exhausting most resources compared to other approximations as reflected by an execution time of 18 μs.

On the other hand, the 3rd order approximations may be classified into rational as in [8] and polynomial as in [1]-a, [1]-c, and [16]. Approximations with d>0 generally provide enhanced error performance, compared to significant execution time edge for polynomial (d=0) approximations, especially when parallel algorithm execution is restricted by the embedded hardware platform. Consequently, the approximation in [1]-a maintains the most favorable computational time/error tradeoff from the available third order polynomials, for the −1≤u≤1 input range.

Previously-reported 2nd order approximations may also be classified similarly, with [1]-b providing the fastest execution time based on a second degree polynomial with modest error performance. In contrast, the proposed approximation (Equation (5)) and the expressions in [10] and [15] are all second order rational approximations of similar forms, with execution times ranging from 3.42 to 3.52 μs, but with a significant variation in their maximum errors. Hence, the comparison is skewed to favor Equation (5), due to its enhanced optimization technique as explained. 

The best performing approximation from each cluster is identified (i.e., Equation (5) for 2nd order, [1]-a for 3rd order, and [14] for 4th order) and, in Section 5, their performance is compared by incorporating them in the proposed flexible arctangent approximation algorithm.

## 4. Proposed Arctangent Approximation Algorithm

Rational approximations provide elegant solution to the arctangent approximation problem, while considering that the error of a given formula depends heavily on the input range and the order of its polynomial(s). As discussed, the nominal input range for rational approximation formulae is typically restricted to −1≤u≤1. Whereas the proposed algorithm in this paper aims to exploit a narrower input range to further optimize the error performance while maintaining favorable execution time. The nominal input range may thus be segmented into a number of intervals (*k*).

This concept of segmentation is the core of the presented algorithm, which combines the main advantages of rational and LUT-based approximation techniques. The rational expression input range is optimized for a narrow interval between u=0 and u=1 (user-defined) within the arctangent function pseudo linear region (i.e., around zero). For that, the absolute input has to be first referred to its 1st octant equivalent using the tangent–cotangent function properties. The narrow defined interval is then concatenated to cover the whole 0≤u≤1 input range, where the determination of the specific segmented interval in which the input lies (e.g., between 9° and 18°) is done using a miniature LUT as detailed below. The estimated angle is finally referred back to its original input octant. This algorithm thus provides flexible error performance that users can adapt based on their application requirements.

### 4.1. Step 1: Input Segmentation Methodology (Input Angle Estimation in −1≤u≤1)

The basic idea of the input segmentation is based on two steps. First, a coarse measure ($\theta_{C}$) of the arctangent is determined by identifying the interval in which the unknown input u=tan(θ) is located. This is achieved by comparing the input to the tangent values of the interval boundaries, which are pre-stored in the LUT (e.g., tan(9°)*,* tan(18°)*,* tan(27°)*,* and tan(36°) within the first octant for *k* = 5) to determine the exact segment in which *u* falls. $\theta_{C}$ is then set as the interval’s upper boundary angle.

Additionally, this operation results in the determination of $\tan\left(\theta_{C}\right),$ which is pre-stored in the LUT. In a second step, a fine value $\theta_{F}$ is then determined within the target narrow interval, which is combined with $\theta_{C}$ to provide an estimate measure $\hat{\theta }$ of the arctangent of the input the range −1≤u≤1:

(6) $$\hat{\theta }=sgn\left(\tan\left(\theta \right)\right)\times \left(\theta_{C}+\theta_{F}\right).$$

However, $\theta_{F}$ may only be determined from the available quantities *u =* $\tan\left(\theta \right),\theta_{C}$, and $\tan\left(\theta_{C}\right)$. For this reason, $tan\left(\theta_{F}\;+\left(45{}^{\circ}/2k\right)\right)$ is first determined using the tangent formula in Equation (7), which is based on the tangent function properties.

(7) $$\tan\left(\theta_{F}+\frac{45{}^{\circ}}{2k}\right)=\tan\left(\left|\theta \right|-\theta_{C}+\frac{45{}^{\circ}}{2k}\right)=\frac{\left|u\right|-\tan\left(\theta_{C}\right)+\tan\left(\frac{45{}^{\circ}}{2k}\right)+\left|u\right|\tan\left(\theta_{C}\right)\tan\left(\frac{45{}^{\circ}}{2k}\right)}{1+\left|u\right|\tan\left(\theta_{C}\right)+\tan\left(\theta_{C}\right)\tan\left(\frac{45{}^{\circ}}{2k}\right)-\left|u\right|\tan\left(\frac{45{}^{\circ}}{2k}\right)}.$$

Note that $\tan\left(\theta_{F}+45{}^{\circ}/2k\right)$ is used instead of $\tan\left(\theta_{F}\right)$ in order to make use of the best linearity of the tangent around the interval’s zero crossing; $\tan\left(\theta_{F}\right)$ is negative and presents more non-linearity than $\tan\left(\theta_{F}+45{}^{\circ}/2k\right)$, which represents the input angle deviation from the interval’s midpoint. That is, $\theta_{F}$ would still range between −9° and 0° for k = 5, but the rational approximation formula only needs to be optimized for half this range because of the adopted shift.

**Figure 3:**
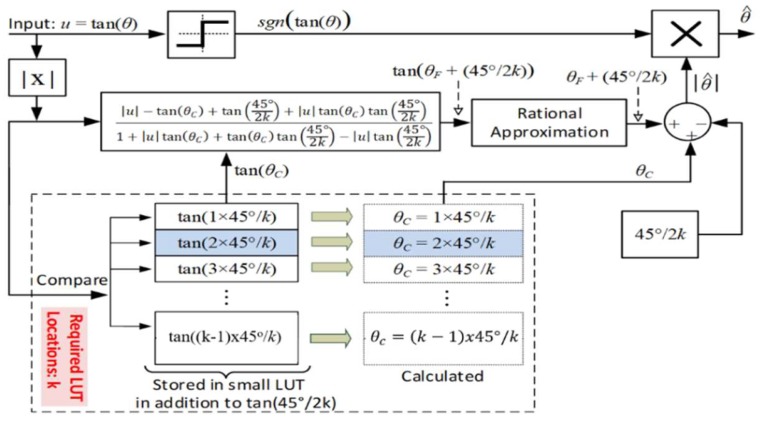
Basic concept the proposed arctangent approximation scheme applicable in the input range  −1≤u≤1.

The value of tan(45°/2k), which is required in Equation (7), may be calculated offline and stored in the LUT for use in the determination of $\tan\left(\theta_{F}+45{}^{\circ}/2k\right)$. Figure 3 illustrates the basic principle of the proposed algorithm for an input range of −1≤u≤1 (i.e., the illustrative block diagram output in Figure 3 is yet to be referred to its original octant in a second step and is not considered as a final algorithm output).

**Figure 4:**
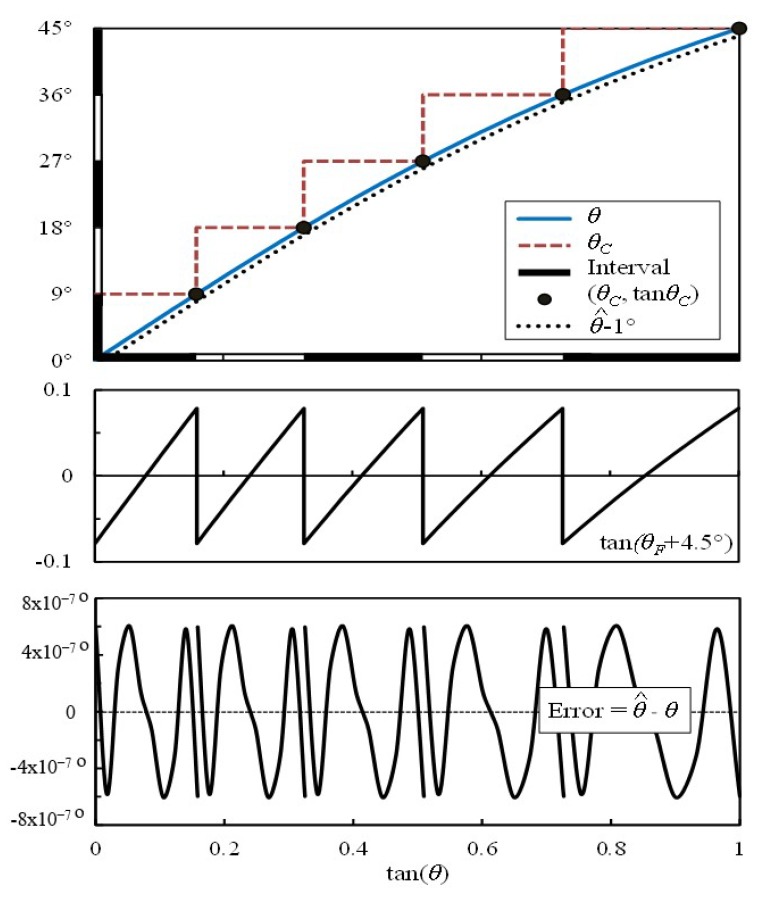
Angle segmentation routine for the proposed algorithm using Equation (5) with k = 5. The estimated arctangent angle is shown shifted down by 1° for clearly distinguishing it from θ.

The pseudo linearity of segmented tangent around zero crossing is clearly illustrated in Figure 4, which also illustrates the various steps leading to the approximation and shows the segmentation of the (0–1) input range into five intervals (k=5) as an example. Notice the mid-interval zero crossing resulting from the adopted shift $\tan\left(\theta_{F}+4.5{}^{\circ}\right)$ instead of $\tan\left(\theta_{F}\right)$. The use of an adequate arctangent approximation formula (for example, Equation (5)), whose coefficients should be re-optimized for the narrow range for the specific chosen number of interval (k) may then be applied to have a precise estimation of $\theta_{F}$ as in Equation (8) for −1≤u≤1 (Equation (8) output is written here in degrees).

(8) $$\theta_{F}=\mathrm{atan}\left(\theta_{F}+\frac{45{}^{\circ}}{2k}\right)-\frac{45{}^{\circ}}{2k}$$

The proposed algorithm thus combines the benefits of both LUT (fast $\tan\left(\theta_{C}\right)$ and *tan (45°/2k)* extraction) and rational (enhanced $\theta_{F}$ optimization accuracy by the narrow range) techniques. The coefficients used in the arctangent approximation formulae in Table 2 are mainly optimized for the input range (–1 to 1). As stated above, these coefficients need to be re-optimized for the new smaller input intervals used in the proposed algorithm in order to exploit the algorithm error reduction capability. Therefore, the minimax error defined in Equations (3) and (4) is re-evaluated based on the selected interval size, in this case, 0<θ<4.5°.

Finally, a numerical example of applying the basic algorithm with an input u = tan(30°) is presented in Table 3 also for k=5. First, the algorithm detects that the selected input falls within the 4th interval (highlighted in bold), calculates $\theta_{C}$, and extracts $tan\left(\theta_{C}\right)\;\mathrm{and}\;\tan\left(45{}^{\circ}/2k\right)$ from the small LUT. Then, $\tan\left(\theta_{F}+45{}^{\circ}/2k\right)$ is calculated using Equation (7) and $\theta_{F}$ is estimated using Equation (8) with the arctangent part approximated using a dedicated rational approximation; in this example case the one in Equation (5). The overall unsigned arctangent approximation $\left|\hat{\theta }\right|$ is then estimated as the sum of $\theta_{C}$ and $\theta_{F}$. Notice that the number of required LUT locations for the algorithm implementation is equal to *k*.

### 4.2. Step 2: Referring the Input to Its Original 360° Value (Generic Algorithm Application)

**Figure 5:**
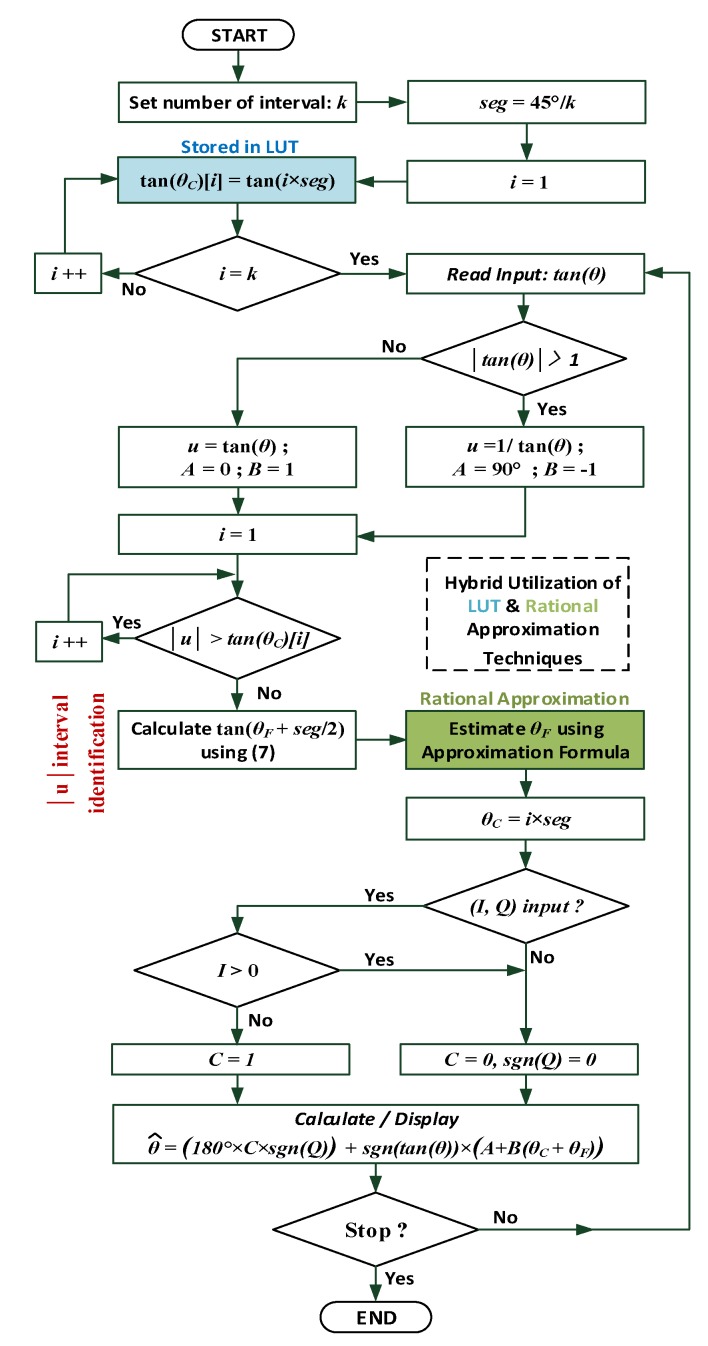
Detailed flowchart of the proposed algorithm. $\theta_{F}$ could be in principle estimated using any re-optimized approximation formula from Table 2.

The basic idea of the proposed algorithm has been thus far described for the input range −1≤u≤1 and corresponding output range (–45°, 45°). This range can be extended to –180° to 180° to refer the input tangent to its original quadrant in case it fell out of this range as illustrated in the flowchart of Figure 5, which explains the detailed steps of implementing the proposed algorithm for a generic number of intervals (*k*).

Initially, the absolute value |tan(θ)| of the input is obtained, referring the signed system input to its 1st quadrant equivalent (0≤θ≤90°). A further range-reduction is achieved by referring this absolute input to its 1st octant equivalent if |tan(θ)|>1. This is done through the cotangent symmetry property:

(9) tan(90°−θ)=cot(θ).

Then, the estimated absolute 1st octant angle $\theta_{C}+\theta_{F}\;$that was calculated in Section 4.1 is referred to its 1st quadrant equivalent by subtracting the 1st octant angle from 90° if |tan(θ)|>1. Thus, extending the application range to −90° ≤ θ ≤ 90° in case of a single-input estimation (route 1 in Figure 1) through using the original input sign. On the other hand, tangent properties are used to extend the generic algorithm implementation range to the full 360° range when two input arguments (route 2 in Figure 1) are accessible, as in Equation (10).

(10) $$atan2\left(I,Q\right)=\left\{\begin{array}{c} \hat{\theta }\;:I\geq 0\\ 180{}^{\circ}\times sgn\left(Q\right)+\hat{\theta }\;:I<0 \end{array}\right\}$$

The flowchart in Figure 5 also uses new variables *A*, *B*, and *C* to present a single, combined illustration of the possible algorithm implementation routes.

Finally, the octant segmentation concept, which is the backbone of this algorithm, has been previously introduced elsewhere using four fixed intervals with linear approximations for each, and was also found to perform well in terms of optimizing power consumption [17]. Yet, the proposed algorithm here is far more powerful in terms of its accuracy, flexibility, and error reduction performance. Note that a major factor for an efficient algorithm implementation is to select an appropriate $\theta_{F}+\frac{45{}^{\circ}}{2k}$ approximation formula that fits the intended application accuracy/time constraints. The presented algorithm requires the use of rational arctangent expressions for $\theta_{F}+\frac{45{}^{\circ}}{2k}$ estimation, which need to be re-optimized for the narrow input range. Any such expression may be theoretically used. However, selecting an adequate formula for the target number of intervals *k* is essential for accurate and fast arctangent approximation. The next section compares the performance of the three rational expressions selected in Section 3 from Equation (5), ref [1]-a. and ref [14] when implemented using the proposed algorithm to benchmark their performance and understand the effect of coefficients re-optimization on each of them.

## 5. Proposed Algorithm Validation

The effect of interval size on the performance of three versions of the proposed algorithm incorporating the three selected rational expressions in Equation (5), ref [1]-a, and ref [14] is studied and compared to evaluate their performance and determine the best suitable expression for the algorithm implementation. The tests carried out are conducted for full 360° range operation, considering signed I and Q inputs for −180°≤θ≤180° to account for the longest algorithm’s execution path scenario. The experimental execution time of all reported approximations has been evaluated using the same eZDSP-F28335 platform, allowing comparison of the required resources under the same test conditions.

### 5.1. Interval Size Effect on the New Algorithm Performance

**Figure 6:**
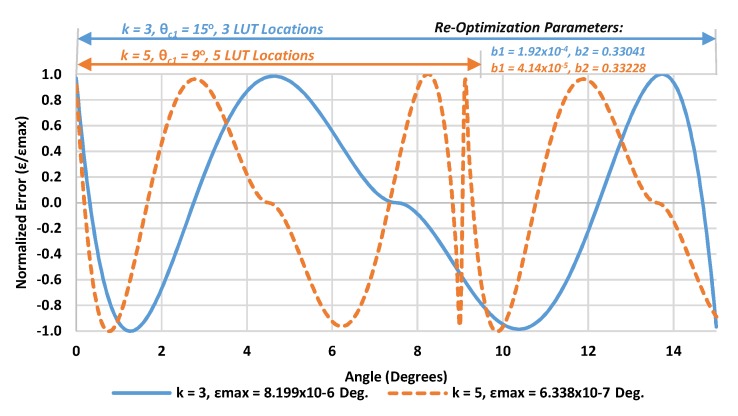
Comparisons of the interval size effect on the maximum error ϵ and required resources for k = 3 and 5 using Equation (5).

The algorithm is first tested to assess the tradeoff between additional accuracy resulting from increasing the number of intervals versus the additional execution time cost. For each interval size and each formula used for $\mathrm{atan}\left(\theta_{F}+45{}^{\circ}/2k\right)$ approximation, the selected rational formulae coefficients have been re-optimized to account for the narrow range approximation, taking into consideration again that the polynomials coefficients shown in Table 2 are only applicable for a single interval implementation range of u = (–1 to 1) (i.e., without input range segmentation). For instance, Figure 6 compares the re-optimization effect in Equation (5) for *k* = 3 and *k* = 5, with the corresponding maximum approximation errors. Clearly, increasing the number of intervals significantly reduces the maximum approximation error, at the expense of an increment in the required resources.

Experimental test results show that, as *k* is increased, significant error reductions with only minimal increments in computational time are observed. That is, the average time cost per added interval for the proposed algorithm using the same DSP platform was practically estimated at ~0.25 μs, regardless of the used approximation formula. Therefore, only a small fraction of the overall calculation time is added for each k increment, since any such addition is only affected by $\theta_{C}$ segment determination loop iterations as illustrated by the flowchart in Figure 5.

In addition, maximum error saturation behavior was observed for very large k values. This is somewhat expected because the basis of the rational approximation is the Taylor expansion of the arctangent function as described in Section 3. Therefore, as *k* increases, segmented arctangent function linearity improves, and the higher power terms of the series become less dominant. Thus, maximum error decay rate is significantly reduced at higher k values when viewed in linear scale, until theoretically reaching zero at an infinitely large number of intervals. 

**Figure 7:**
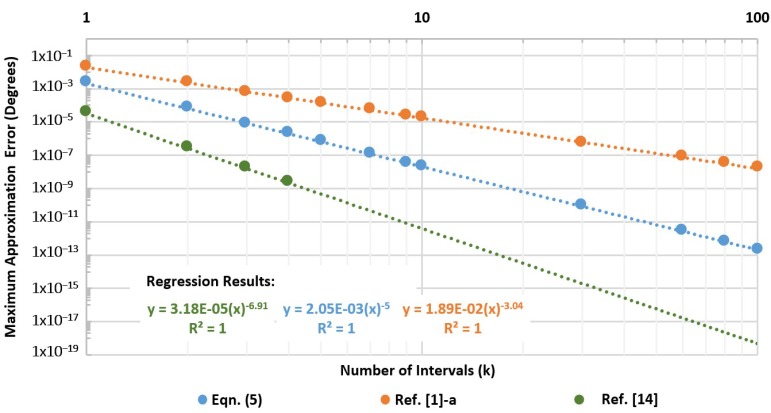
Maximum error vs. number of intervals (k) for different rational approximation formulae.

In contrast, a consistent error decay is observed under logarithmic scale when plotting the maximum error vs. number of intervals (k). The same behavior is observed for the three selected expressions from ref [1]-a, ref [14], and Equation (5), however, with unique error decay slopes as presented by Figure 7. The direct result inferred from this figure is the superior error-reduction performance with increasing number of intervals for the approximation from ref [14] due to its fourth-order rational nature.

Consequently, a numerically derived relation through non-linear fitting presents an elegant mathematical form in Equation (11) of a generic equation relating both variables ($\varepsilon_{max,k}$ and k) for estimating the maximum error at a given interval size based on any compatible rational approximation formula from Table 2.

(11) $$\varepsilon_{max,k}\approx \left(\varepsilon_{max,1}\right)\;k^{-\gamma }$$

The constant $\varepsilon_{max,1}\;$represents the maximum error for k=1 ($\theta_{C}=45{}^{\circ},\;0\leq u\leq 1)$, whereas γ is the unique error decay rate for a specific approximation with k. 

Equation (11) is very useful in that it can be used to pre-estimate the required intervals for a given approximation to achieve a specific application target error for further resources optimization. Namely, it is ideally sufficient for an approximation of similar form to be optimized for two independent intervals only (*k* = 1 and any other *k* value), and to use these optimization results to obtain the expression’s decay constant γ, leading to a powerful tool for quick interval size requirement assessment for any target application maximum error. That is, one can set a target error for its application, determine the required *k* from Equation (11), and finally re-optimize the approximation coefficients used for that particular interval size.

### 5.2. Performance Comparison of the Three Selected Rational Formulae for Algorithm Implementation

It is essential to take both accuracy and execution time performance into account when selecting adequate approximation from the available candidates for $\theta_{F}$ estimation using the novel algorithm. Setting a very low target error increases the required accuracy, corresponding to adjusted number of intervals *k,* at the expense of higher algorithm’s execution time.

To illustrate the effect of this tradeoff on different approximation formulae, a test application case is established with a target error of $6.338\times 10^{-7}{}^{\circ}.$ The resources to achieve it for the three selected expressions are compared. This target error is selected to reflect high performance requirements in some applications, where the considered number itself is the maximum error achieved by Equation (5) at k=5. It should be noted that the minimum achievable target error is also dependent on the used hardware board limitations (i.e., the number of bits supported at the output, and the mantissa adjustment flexibility).

#### 5.2.1. Fourth Order Approximation

The number of intervals to achieve the target error using the proposed algorithm with the rational approximation from ref [14] for $\theta_{F}$ estimation is only k=2. This shows the superior error performance of this formula. However, the required execution time to achieve this error is 27 μs. This is expected due to the large number of bit operations required for the 4th order rational calculation as evident from Table 2’s execution time comparison. This execution time includes the 18 μs for the rational formula itself, in addition to the time required for the algorithm execution, as well as the ~0.25 μs increment per k.

#### 5.2.2. Third Order Approximation

The required number of intervals for achieving the same error using the selected 3rd order approximation from ref [1]-a is significantly larger at k=30, increasing the overall algorithm’s execution time to 14.40 μs. The significance of this increment is easily viewed when compared to the execution time of the standalone formula in Table 2 as 0.58 μs. The computational time edge for this formula is thus severely affected when combined with the proposed algorithm when higher accuracy targets are required. 

#### 5.2.3. Second Order Approximation

**Figure 8:**
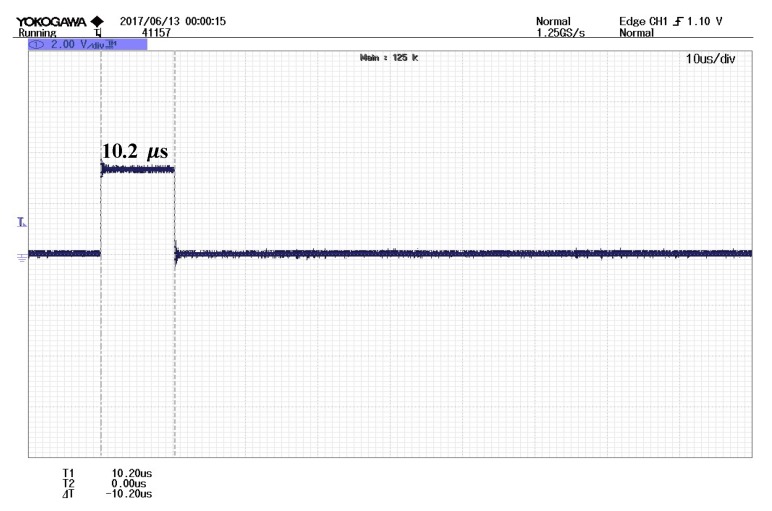
Practical result of the proposed algorithm execution time with *k* = 5 using Equation (5).

The required number of intervals in this case is moderate at k =5. The execution time for the formula itself is 3.50 μs from Table 2. The overall time including the algorithm and the intervals addition is 10.20 μs (see Figure 8). This indicates a favorable execution time edge for the proposed algorithm when combined with this formula at higher accuracies. 

#### 5.2.4. Performance Analysis

Results for the three formulae are summarized by Table 4 for the set target error. As established, the approximation in ref [14] had a superior error performance in Table 2, far ahead when compared to other candidate formulas. However, the proposed algorithm exploits different approximations ability to achieve miniature errors, adding the timing performance as a decisive selection factor. Table 4 indicates that the lower error edge for the 4th order formula in ref [14] is overshadowed by excessive execution time for the set target error. In contrary, the approximation in ref [1]-a clearly loses its superior timing edge when higher accuracy is required, while using many LUT locations, which ultimately defies the algorithm’s purpose and makes it similar to conventional, memory oriented, LUT techniques. Equation (5), on the other hand, balances both factors and results in a favorable tradeoff. As a result, it is the selected formula to be generically used with the proposed algorithm for fast and efficient arctangent approximation in real-time applications.

## 6. Conclusions

This paper presented a novel algorithm for full-range fast arctangent approximations, combining the advantages of existing rational and LUT-based techniques. The algorithm is developed to suit generic applications with user-defined accuracy requirements, providing a high level of flexibility. Namely, the algorithm overcomes the rational approximations main limitation of providing a fixed minimized-maximum error, and creates room for further error range optimization in combination with small LUTs. The new algorithm’s operating principle is based on decomposing the input tangent into a fixed $\theta_{C}$ and varying $\theta_{F}$ values by segmenting the first octant referenced angle into equally spaced intervals, hence restricting the varying input range of the rational approximation formula to the pseudo linear portion of the arctangent function, and improving the overall approximation accuracy at a moderate additional resources expense. Rational approximations from the literature were also thoroughly evaluated compared to a new 2nd order introduced expression in terms of their performance and complexity. The new formula was shown to be more adequate for the algorithm application in terms of execution time vs. accuracy tradeoff when high accuracy margins are required by the target application. Reduced maximum errors were obtained by using rational expression’s minimax re-optimization for each interval size rather than using the generic input −1≤u≤1 optimization range. The presented results were validated through experimental implementation using the eZDSP-F28335 platform. Finally, the contributions made in this work are not only limited to the proposed arctangent approximation algorithm but extend to establishing a common evaluation index to the existing rational approximation formulae. In addition to the possible extension of the input segmentation methodology to other similar applications.

## Figures and Tables

**Table 1:** Qualitative Comparison of Arctangent Approximation Methods.

Technique	Advantages	Limitations
**CORDIC**	Requires minimal hardware resources. Can theoretically achieve any target minimum error.	High execution time for high-accuracy applications because of the large number of required iterations.
**LUT**	Very fast execution. Enhanced accuracy using interpolation.	Excessive memory requirements for high-accuracy applications.
**Rational**	High Accuracy *. Efficient Implementation *.	Constant maximum error per expression (non-adaptive). Computationally expensive for higher-order expressions.

* Depends on the used approximation formula.

**Table 2:** Arctangent rational approximations, their practical execution time on the eZDSP platform, and their maximum errors.

Work	atan(u) Approximation (rad) for −1≤u<1	Maximum Error (°)	Time (μs)
[14]	$u\left(\frac{1+0.372003u^{2}}{1+0.703384u^{2}+0.043562u^{4}}\right)$	0.0030	18.00
[8]	$\frac{\pi }{2}\left(\frac{u}{1+u}\right)\left(\frac{\alpha +u+u^{2}}{1+\alpha u+u^{2}}\right);\;\alpha =\frac{1+\sqrt{17}}{8},\;range:\;0\leq u<\infty$	0.0081	6.60
Present, Equation (5)	$\frac{u}{1+0.0443\left|u\right|+0.2310u^{2}}$	0.0777	3.50
[1]-a *	$u\left(\frac{\pi }{4}+\left(1-\left|u\right|\right)(0.2447+0.0663\left|u\right|\right)$	0.0862	0.57
[15]	$u\left(\frac{4.66+8\left|u\right|}{5+6\left|u\right|+5.1u^{2}}\right)$	0.2000	3.52
[1]-b	$u\left(\frac{\pi }{4}+0.273\left(1-\left|u\right|\right)\right)$	0.2138	0.54
[10]	$\frac{u}{1+0.28125u^{2}}$	0.2632	3.42
[1]-c	$u\left(\frac{\pi }{4}+0.186982-0.191942u^{2}\right)$	0.2833	1.91
[16] **	$\frac{\pi }{4}u\left(1+0.23175\left(1-u^{2}\right)\right)$	0.3502	1.92

* Reference [1] includes 3 different approximations, titled here a, b, and c; ** The reported equation is re-written here in the same format as other formulas.

**Table 3:** Numeric Application example of the proposed algorithm.

k= 5 seg=45°÷k=9°		
***i***	$\tan\left(\theta_{C}\right)\left[i\right]$	$\theta_{C}=i\times seg$
1	0.1584	9°
2	0.3249	18°
**3**	**0.5095**	**27°**
**4**	**0.7265**	**36°**
5	1.0000	45°
**Example of atan (*u*) Estimation Using Proposed Method with Equation (5).**		
**Input:**		$u=\tan\left(\theta \right)=1/\sqrt{3}\approx 0.5774\;\left(\theta =\;30.0000000{}^{\circ}\right)$
**STEP 1:** $0.5095<\tan\left(\theta \right)<0.7265\to \tan\left(\theta_{C}\right)\approx 0.72658\;\mathrm{and}\;\theta_{C}=36{}^{\circ}\;$		
**STEP 2:** calculate $\tan\left(\theta_{F}+4.5{}^{\circ}\right)$		**STEP 3:** Estimate $\theta_{F}+\frac{45{}^{\circ}}{2k}$ using adequate formula, e.g., Equation (5).
$\tan\left(\theta_{F}+4.5{}^{\circ}\right)=\frac{\left|u\right|-\tan\left(\theta_{C}\right)+\tan\left(4.5{}^{\circ}\right)+\left|u\right|\tan\left(\theta_{C}\right)\tan\left(4.5{}^{\circ}\right)}{1+\left|u\right|\tan\left(\theta_{C}\right)+\tan\left(\theta_{C}\right)\tan\left(4.5{}^{\circ}\right)-\left|u\right|\tan\left(4.5{}^{\circ}\right)}\approx -0.02618592$		$x=\theta_{F}+4.5{}^{\circ}\approx \frac{180{}^{\circ}}{\pi }\times \frac{\tan\left(x\right)}{1+b_{1}\left|\tan\left(x\right)\right|+b_{2}\tan^{2}\left(x\right)}$ $b_{1}=4.14\times 10^{-5}$; $b_{2}=0.33228$ $\theta_{F}\approx -5.9999994{}^{\circ}$
−1≤u≤1 **Range Output:**		$\hat{\theta }=\theta_{C}+\theta_{F}\approx 36{}^{\circ}-5.9999994{}^{\circ}=\;30.0000006{}^{\circ}$

**Table 4:** Test case: Performance assessment of the three $\theta_{f}$ approximation candidates based on a common target error.

Work	Ref. [14]	Equation (5)	Ref. [1]-a
Test Case Target Error	$6.338\times 10^{-7}$ Degrees		
Required Intervals (*k*)	2	5	30
Computational Time (μs)	27.00	10.20	14.40
Required LUT locations	2	5	30

## Dedication

This work is dedicated to its first author and main idea contributor, Professor Mohieddine Benammar, who passed away during the last preparation stages of this manuscript. He bravely battled cancer for 6 years and did not allow it to limit his innovation until the last of his days. His legacy will always remain as a guide to his fellow scholars and students in his various fields of research.
